# A deep dive into the diversity of the *Aspergillus* community in the lakes of northern Iran

**DOI:** 10.3389/fpubh.2026.1759055

**Published:** 2026-03-05

**Authors:** Meysam Kor, Mohammad Taghi Hedayati, Mahdi Abastabar, Iman Haghani, Mojtaba Nabili, Abolfazl Saravani, Javad Javidnia, Joao Brandão, Maryam Moazeni

**Affiliations:** 1 Student Research Committee, Mazandaran University of Medical Sciences, Sari, Iran; 2 Department of Medical Mycology, School of Medicine, Mazandaran University of Medical Sciences, Sari, Iran; 3 Invasive Fungi Research Center, Communicable Diseases Institute, Mazandaran University of Medical Sciences, Sari, Iran; 4 Department of Medical Laboratory Sciences, Sar.C., Islamic Azad University, Sari, Iran; 5 Departamento de Saúde Ambiental, Instituto Nacional de Saúde Doutor Ricardo Jorge, Lisbon, Portugal; 6 CE3C– Centre for Ecology, Evolution and Environmental Changes, Faculdade de Ciências da Universidade de Lisboa, Lisbon, Portugal

**Keywords:** AFST, *Aspergillus*, fungi, Iran, lake mycobiome

## Abstract

**Introduction:**

This study aimed to evaluate the mycological quality of soil and water from recreational lakes in the Mazandaran region, focusing on the diversity and abundance of *Aspergillus* species and their antifungal susceptibility profiles.

**Methods:**

Sampling was conducted at 42 stations across 7 selected lakes in Mazandaran province during the bathing season (summer months of 2023). Three different types of samples, namely dry soil, sediment (sediment), and water were collected at each station in accordance with the standard operational procedure (SOP) established by the leaders of the Mycosands project. The isolated *Aspergilli* were identified using polymerase chain reaction–restriction fragment length polymorphism (PCR-RFLP). Antifungal susceptibility testing was performed following the guidelines set by the Clinical Laboratory Standards Institute.

**Results:**

Of 713 *Aspergillus* isolates, 353 (49.58%) were obtained from soil, 272 (35.8%) from sediment, and 87 (12.2%) from water samples. The *Aspergillus* section *Terrei* exhibited the highest abundance (*n* = 295, 41.37%), particularly in Elimalat (288 CFU/mg/mL), which showed the most diverse range of species. Soil samples revealed *A.* sec. *Terrei* as the most prevalent (273 CFU/mg) whereas sediment and water samples showed lower loads of *Aspergilli,* with the section *Nigri* identified most frequently (176 CFU/mg and 105 CFU/mL, respectively). Elevated minimum inhibitory concentrations (MICs) against itraconazole and voriconazole were observed in 3.22 and 1% of isolates, respectively.

**Conclusion:**

Summer conditions may have contributed to increased fungal contamination due to increased human activity and organic waste. Therefore, prioritizing microbial contamination control and improving hygienic awareness among visitors remain vital. However, seasonal sampling and section-level identification represent limitations that should be addressed in future studies.

## 1 Introduction

Lakes, as vital reservoirs of surface water, play a significant role in maintaining ecological balance, supporting wildlife, and providing water resources for both human consumption and agriculture (1). However, these environments are often exposed to environmental contamination, which can facilitate the proliferation of pathogenic microorganisms, including opportunistic fungi. This contamination not only poses risks to human health but also threatens the stability of aquatic ecosystems (2). Recent studies suggest that fungi in coastal environments can enter these ecosystems through rainfall, floods, and decaying plant debris (3). The presence of fungal species, such as dermatophytes, *Aspergillus*, and *Candida* in recreational waters and sand has become a subject of increasing concern, as it can impact the health and safety of beachgoers, particularly during the summer months when these areas are most heavily utilized (2, 4). Continuous exposure of tourists to contaminated sand can increase the risk of fungal skin and respiratory infections, particularly in immunocompromised individuals (5). Hence, monitoring water quality is essential for protecting public health and preventing waterborne diseases. Traditionally, research has primarily focused on pathogenic bacteria and viruses, particularly those associated with gastrointestinal disorders (4, 6). However, the investigation of fungal communities in coastal beach sand and recreational waters is still insufficiently explored, despite their possible effects on human health. Since (7), the World Health Organization (WHO) has emphasized the significance of this issue; however, the majority of research studies has predominantly focused on bacterial contamination (8). Recently, there has been an increasing interest in the fungi found in beach sand, particularly since certain identified species are recognized to be linked with conditions such as dermatomycoses, allergic reactions, otitis, and deep mycetoma (9). The “Mycosands” Working Group, a collaboration among European Confederation of Medical Mycology, European Society for Clinical Microbiology and Infectious Diseases, and The International Society for Human & Animal Mycology, conducted a study on the presence of fungi in sand and water samples collected from 13 countries, mainly across Europe (10). However, extensive distribution data concerning coastal fungi are still lacking in many regions worldwide. As a result, there is limited information on the prevalence of fungal species along coastlines, particularly those surrounding the lakes of Iran. Notably, the diversity and prevalence of medically significant fungi found in the beach sand and water of the Caspian Sea and the Persian Gulf have previously been assessed by the same research group (11, 12).

Mazandaran province, located along the southern coast of the Caspian Sea, hosts several ecologically significant lakes (13). The lakes in Mazandaran are situated at elevations between 400 and 1,200 m above sea level, extending across the forested and mountainous regions of northern Iran (7). These lakes serve as vital habitats for a wide range of microbes while also contributing to tourism and local economies (14).

These findings reinforce the need for continuous microbial monitoring of sandy beaches and recreational waters, particularly in areas affected by environmental pollution. Accordingly, this study reported the mycological quality of soil and water from the recreational lakes in the Mazandaran region, focusing on the diversity and abundance of *Aspergilli* and their antifungal susceptibility profiles.

## 2 Materials and methods

### 2.1 Study design

**Figure 1:**
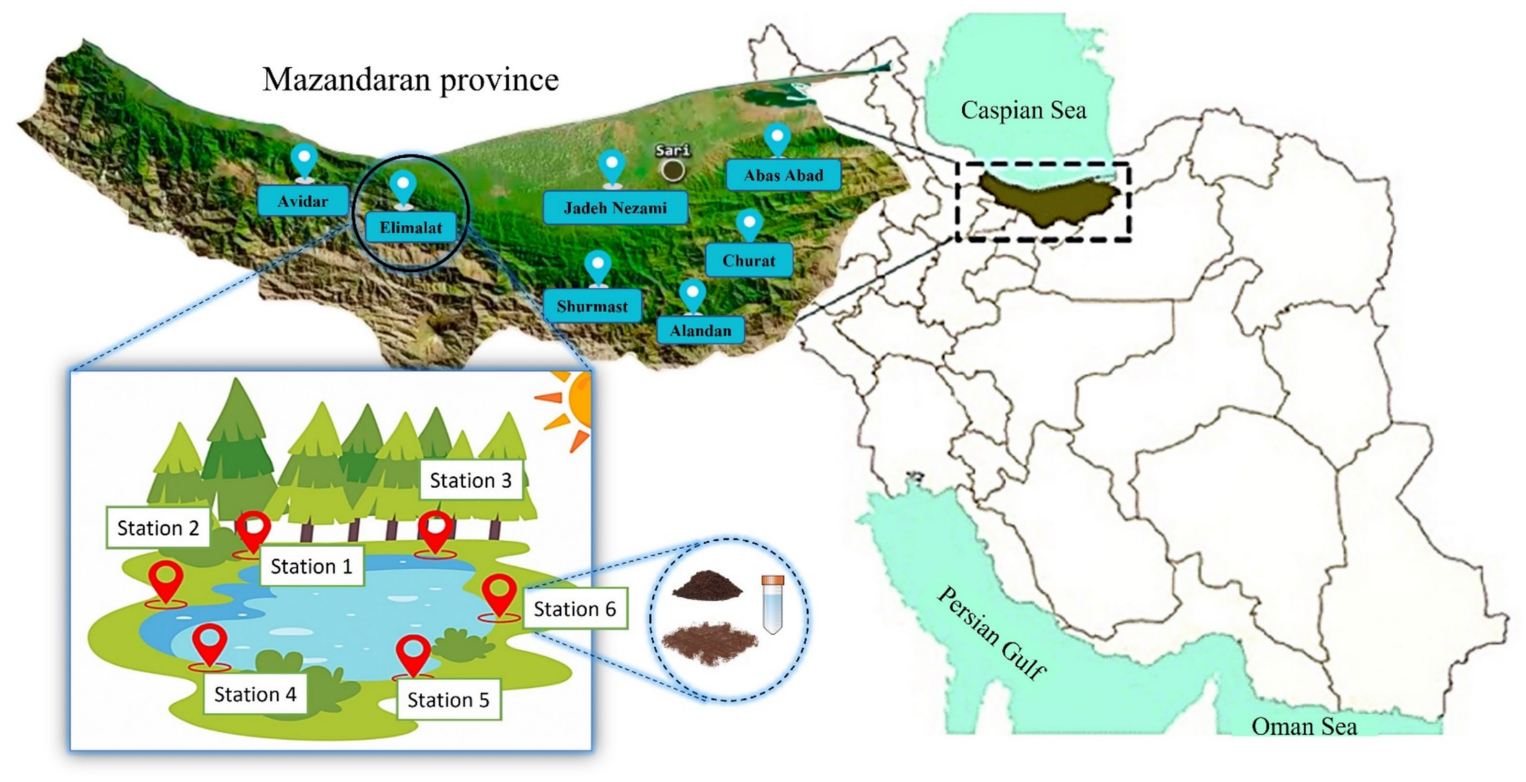
The lakes selected for sampling are situated in the Mazandaran province, which is positioned along the Caspian Sea. Each lake was divided into six stations, and from each station, three different samples were collected. Overall, 42 soil, 42 sediment, and 42 water samples were collected from 42 stations during July and September 2023.

This study was conducted at seven selected lakes in Mazandaran province located in northern Iran, which had mild and humid climate during the summer of 2023. The lakes included Abbas Abad, Alandan, Churet, Jedeh Nezami Elimalat, Avidar, and Shurmast. For each lake, the number of sampling stations was determined based on the lake size, tourism characteristics, and visitor density along the shoreline, resulting in a total of 42 stations. All these lakes are visited by people all around Iran during the bathing season, which typically spans late spring through the entire summer. Sampling was conducted during the bathing season, focusing particularly on the most visited months, specifically July and September. Figure 1 depicts the exact locations of the studied lakes within the Mazandaran province. Some parameters, such as temperature and humidity, were recorded on the sampling day. The measurements were recorded on-site and were not sourced from meteorological data. The study was approved by the Ethics Committee of Mazandaran University of Medical Sciences (Approval No. IR. MAZUMS. REC.1402.312).

### 2.2 Sample collection

Sampling was conducted at each of the 42 stations across 7 lakes in Mazandaran province during the summer of 2023, specifically in July and September 2023. Three different types of samples were collected at each station: dry soil from the central area of dry shoreline at a depth of 10 cm, sediment (sediment) samples from the lakebed at a depth of 1 m, and water samples from a depth of 20 cm. Sampling was performed once a month, exclusively on sunny days between 10 a.m. and 3 p.m. to minimize temperature variability according to the standard operational procedure (SOP). This SOP was adopted from Sabino et al. (15) and was agreed upon by the leaders of the Mycosands project (16). Overall, 42 soil, 42 sediment, and 42 water samples were collected from 42 stations during July and September 2023. The soil samples were aseptically collected in sterile 50 mL Falcon flasks and later homogenized in sterile plastic containers.

The total weight of the final samples including dry soil and sediment was approximately 200 g. Seawater samples were collected aseptically in two sterile 500 mL vessels (Golias, Ljubljana, Slovenia). All samples were labelled and transported to the laboratory in a cooler within 2 h of sampling, as described by Sabino et al. (15).

### 2.3 *Aspergillus* load determination and species identification

Each collected soil sample (approximately 40 g, maintained at natural moisture without oven drying) was homogenized by agitation for 30 min at 100 rpm. Subsequently, 0.2 mL of the resulting suspension was inoculated onto the following media: (a) Sabouraud’s dextrose agar (SDA; Scharlau, Madrid, and Spain) plates supplemented with chloramphenicol, (b) SDA plates supplemented with chloramphenicol/itraconazole at a concentration of 4 mg/L, and (c) SDA plates supplemented with chloramphenicol/voriconazole at a concentration of 2 mg/L. To achieve the study objectives, antifungal-supplemented media were utilized for the initial screening and identification of potentially resistant strains. The cultured petri dishes were incubated at 30 °C for up to 3 weeks, and fungal colonies were monitored regularly. Water samples (0.2 mL each) were similarly cultured after brief agitation. The fungal load was assessed quantitatively by counting colony-forming units (CFU)/g and (CFU)/mL for soil and water samples, respectively. Initial fungal identification was performed through both microscopic and macroscopic assessments, utilizing the KOH direct examination test. The identification process was further supported by employing selective media such as Czapek agar (Himedia, Mumbai, Maharashtra, India). Microscopic morphology was assessed using wet mounts stained with the lactophenol cotton blue staining method.

### 2.4 Molecular identification of the recovered *Aspergillus* isolates

To confirm the morphological findings, molecular identification of the isolates was conducted using PCR-RFLP that targeted the beta-tubulin gene. DNA was extracted from colonies cultivated for 24–48 h on SDA medium through a phenol/chloroform extraction method (17). PCR amplification that targeted the beta-tubulin gene region was conducted using specific primers: Bt-F (5′-GGTAACCAAATCGGTGCTGCTTTC-3′) and Bt-R (5′-ACCCTCAGTGTAGTGACCCTTGGC-3′). The amplification was performed on a Corbett Research thermal cycler, model CG1-96 (Sydney, Australia). The PCR reactions were conducted in a 50 μL volume containing 25 ng of template DNA, 25 pmol of each primer, and 12.5 μL of reaction master mix (Amplicone, The Netherlands). The BT2 amplification involved an initial denaturation step at 95 °C for 5 min, followed by 35 cycles of 95 °C for 45 s, 60 °C for 120 s, and 72 °C for 60 s, with a final extension at 72 °C for 7 min. The resulting PCR products were digested with the restriction enzyme *Alw*I (*BspP*I) (Life Technologies, Carlsbad, CA, USA) and subsequently electrophoresed for section-level identification according to previously established protocols (18).

### 2.5 Antifungal susceptibility tests for isolates grown on antifungal-supplemented media

Antifungal susceptibility testing was conducted following the Clinical and Laboratory Standards Institute (CLSI) guidelines M38 for *Aspergillus* isolates grown on antifungal-supplemented media. This screening may be considered as a limitation of our study; however, since the aim of the study was to select the isolates with high MICs, the preliminary screening was designed to include only isolates grown on antifungal-supplemented media for antifungal susceptibility testing (AFST). The document M59 was also referred for antifungal testing without certain breakpoints (19, 20). The susceptibility test assay was performed only for the *Aspergillus* isolates recovered from ITZ/VRZ-supplemented media. The antifungal agents, itraconazole (ITZ) and voriconazole (VRZ), were prepared in a standard RPMI 1640 medium (Sigma-Aldrich, St. Louis, MO, USA) and buffered to pH of 7.0 using 0.165 M 3-(N-Morpholino) propane sulfonic acid (MOPS, Sigma Chemical Co.), combined with L-glutamine and without bicarbonate, to yield a concentration that was twice their standard level. The antifungal agents were distributed in 96-well microdilution plates (Nunc, UK) at final concentrations ranging from 0.016 to 16 μg/mL for both ITZ and VRZ. The microplates were incubated at 35 °C for 48 h. The minimum inhibitory concentrations (MICs) were determined visually at 100% inhibition of growth after 24 h of incubation at 35 °C for tested drugs compared to positive controls. *Hamigera insecticola* (previously identified as *Paecilomyces* var*iotii*) (ATCC 22319) was used as the quality control isolate and was included on each day of testing.

### 2.6 Statistical analyses

Statistical analysis was conducted using Microsoft Excel (version 2013) and SPSS for Windows (version 22; SPSS Inc., Chicago, IL, USA). Descriptive statistics were applied to assess the antifungal susceptibility data, which were reported as minimum inhibitory concentrations (MICs), including MIC_50_ (MIC inhibiting 50% of isolates) and MIC_90_ (MIC inhibiting 90% of isolates). Associations with environmental factors were analyzed using ANCOVA and Mann–Whitney *U* tests. A *p*-value of less than 0.05 was considered statistically significant.

## 3 Results

### 3.1 *Aspergillus* species load (CFU/mL) in soil and water

A total of 126 samples from 42 stations were collected across seven lakes. Soil, sediment, and water samples were gathered and analyzed for *Aspergillus* species using morphological characteristics and PCR-RFLP methods at the medical mycology laboratories of Mazandaran University of Medical Sciences, Sari, Iran. Totally, 713 *Aspergillus* isolates were identified. Among 353 (49.58%) isolates were recovered from soil, 272 (35.8%) isolates from sediment, and 87 (12.2%) isolates from water samples.

The lake Elimalat (288 CFU/mg/mL, *A.* sec. *Terrei* was the most prevalent), Jadeh Nezami (190 CFU/mg/mL, *A.* sec. *Flavi* was the most prevalent), and the lake Avidar (181 CFU/mg/mL, *A.* sec. *Nigri* was the most prevalent), were the lakes with the highest reported *Aspergilli* load. A great proportion of the *Aspergillus* species were identified from soil (538 CFU/mg) with the prevalent species belong to section *Terrei* (237 CFU/mg) whereas sediment revealed lower load of *Aspergilli* (434 CFU/mg) with the predominant species belong to *A.* sec. *Nigri* (176 CFU/mg). However, the difference in fungal loads with various reserves was not significant (*p* < 0.05). Water samples comprised 151 CFU/mL, which was significantly lower that soil and sediment samples (*p* < 0.05). Among the various species, *A.* sec. *Nigri* was the major species recovered from water samples (105 CFUs/mL). According to morphologic identification, which was the primarily method of identification for CFU counting, *A.* sec. *Nigri* (458 CFU/mg/mL) was reported as the section with the highest abundance followed by species belong to *A*. sec. *Terrei* (419 CFU/mg/mL), and section *Fumigati* (195 CFU/mg/mL). Table 1 shows the distribution of *Aspergillus* species recovered from soil, sediment, and water samples across seven studied lakes.

**Table 1:** The distribution of *Aspergillus* species recovered from soil, sediment, and water content across seven studied lakes.

**Lake Name**	**Sample type**	**Fungal load (Average CFUs/mg/ml)**						
		***A. s*ec. *Flavi***	***A.* sec. *Fumigati***	***A.* sec. *Nigri***	***A.* sec. *Terrei***	***A.* sec. *Nidulanti***	**Unknow**	**Total**
**Elimalat**	Soil	3	2	22	212			
	Sediment	3	2	10	23			288
	Water		2	7	2			
**Churet**	Soil	5		10	10			
	Sediment	5	2	12	52			114
	Water	5		10	3			
**Alandan**	Soil	8	2	20	2		5	
	Sediment	13	2	35	12			121
	Water	7		15				
**Shourmast**	Soil	17		27				
	Sediment	8		47	13			151
	Water	7		30	2			
**Abbasabad**	Soil	3		28	8			
	Sediment	2		15	2			78
	Water	3		17				
**Avidar**	Soil			32				
	Sediment	2	22	30	63	5		181
	Water	2		18	7			
**Jade Nezami**	Soil	77	2	38	5			
	Sediment	22	2	27	3			190
	Water	3	3	8				
	**Total Fungal load (Average CFUs/mg/ml)**						**Total**	
**All Lakes**	**Soil**	**113**	**6**	**177**	**237**	**-**	**5**	**538**
	**Sediment**	**55**	**30**	**176**	**168**	**5**	**-**	**434**
	**Water**	**27**	**5**	**105**	**14**	**-**	**-**	**151**
**Total**		**195**	**41**	**458**	**419**	**5**	**5**	**1123**

*Aspergillus* sec. *Nigri* (458 CFU/ mg/ ml) was reported as the section with the highest abundance followed by species belong to section *Terrei* (419 CFU/ mg/ ml), and section *Fumigati* (195 CFU/ mg/ ml).

### 3.2 Species identification of *Aspergilli* and the role of environmental parameters

**Figure 2:**
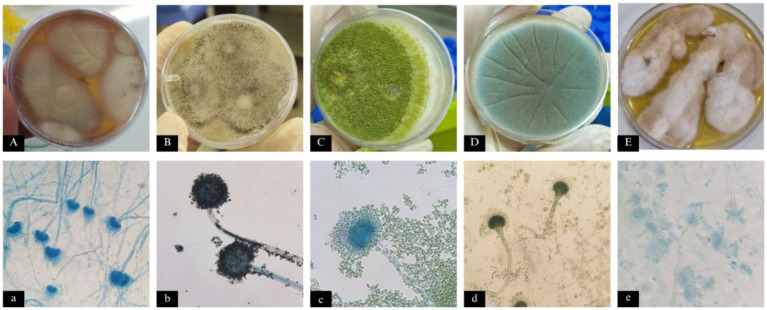
Microscopic and macroscopic features of fungal species isolated from different samples: **(A, a)**: *Aspergillus* section *Terrei*, **(B, b)**: *A.* sec. *Nigri*, **(C, c)**: *A.* sec. *Flavi*, **(D, d)**: *A.* sec. *Fumigati*, and **(E, e)**: *A.* sec. *Nidulanti.*

Morphologic identification of the isolates revealed that *A.* sec*. Terrei* exhibited the highest abundance with 285 isolates (40%), followed by *A.* sec. *Nigri* with 270 isolates (37.9%); *A.* sec. *Flavi* with 117 isolates (16.4%), *A.* sec. *Fumigati* with 23 isolates (3.2%), and *A.* sec. *Nidulantes* with 2 isolates (0.3%). About 16 strains could not be classified into any of the most common *Aspergillus* sections, morphologically. Figure 2 illustrates the microscopic and macroscopic features of selected fungal species isolated from different kind of samples.

**Figure 3:**
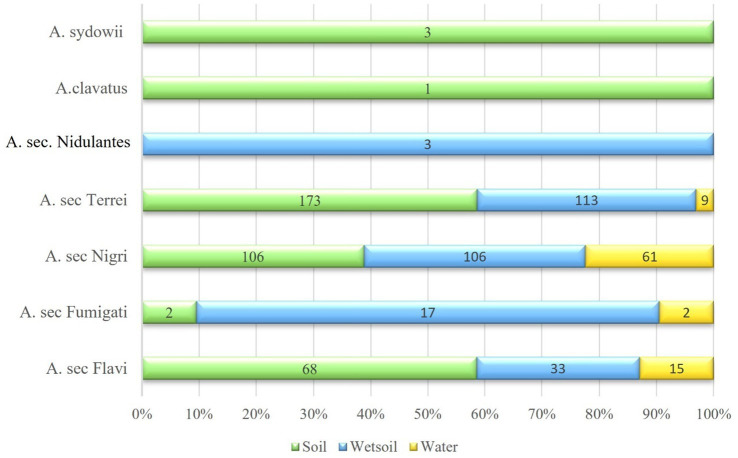
Overall distribution of *Aspergillus* sections in soil, sediment, and water samples from July to September 2023. A great proportion of the *Aspergillus* species were identified from soil (353, 49.58%) with the prevalent species belong to *Aspergillus* section *Terrei,* which accounts for 295 isolates (41.37%).

**Figure 4:**
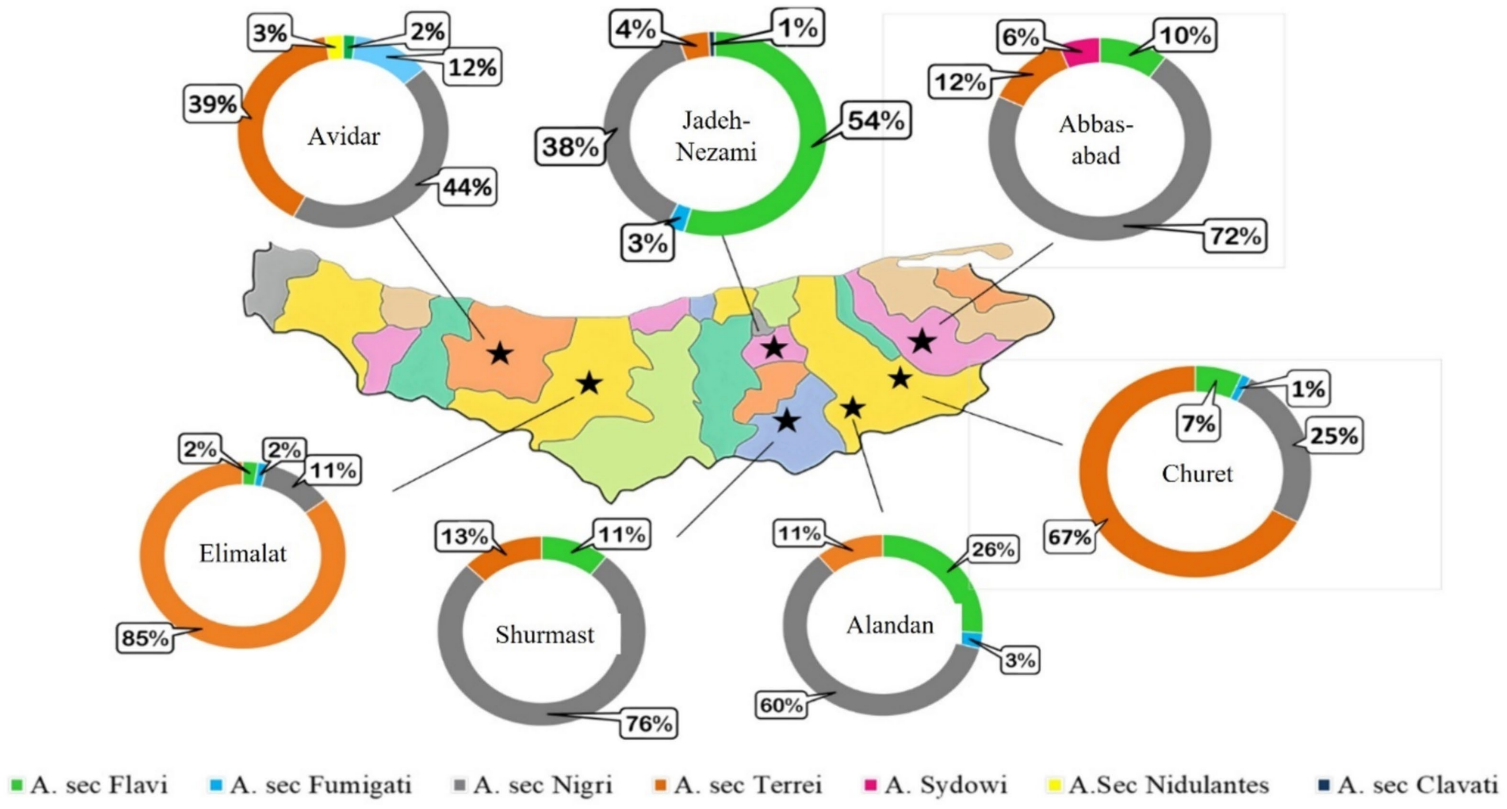
Distribution of *Aspergillus* sections across lakes in Mazandaran province based on geographical locations from July to September 2023. The Elimalat lake with the highest concentration of CFUs was predominantly inhabited by *A.* sec. *Terrei* (85%).

PCR-RFLP results provided more accurate data about morphologic identification and also enabled identification of some unknown strains. However, three strains remained unknown using this technique. The molecular identification of the isolates revealed that *A.* sec. *Terrei* displayed the greatest abundance, with a total of 295 isolates (41.37%), predominantly found in soil (wet/dry) (173 isolates, 58.64%). However, *A.* sec. *Nigri* accounted for 273 isolates (38.3%) and was equally dominant in both soil and sediment (106 isolates each), *A.* sec. *Flavi* accounted for 116 isolates (16.3%), while *A.* sec. *Fumigati* accounted for 21 isolates (2.95%) was primarily detected in sediment (17 isolates, 80.9%). Both *A.* sec. *Nidulantes* with 3 isolates (0.4%) and *A.* sec. *Clavati* with 1 isolate (0.1%) exhibited a lower frequency of *Aspergillus* sections (Figure 3). The three isolates (0.42%) that remained unknown even after PCR-RFLP identification were identified as *A. sydowii* after performing *TUB*-sequencing technique. Figures 3 and 4 depict the distribution of *Aspergillus* sections across lakes in Mazandaran province based on geographical locations and also on the sample source. The Elimalat area with the highest concentration of CFUs was predominantly inhabited by *A.* sec. *Terrei*, accounting for 85%. The primary species identified from Churet and Avidar was also *A.* sec. *Terrei*, with recovery rates of 67 and 39%, respectively. The average temperature for air, soil, and water were recorded at 24.5 °C, 20.2 °C, and 20.7 °C, respectively. Additionally, the average humidity was 45.8%. These results represent mean values obtained from the three specified lake locations during both sampling rounds. The most common species found in Jade Nezami Lake was *A.* sec. *Flavi*, which constituted 54% of the isolates. The average temperature for air, soil, and water were reported as 33.5 °C, 26.5 °C, and 27.5 °C, respectively, and the mean humidity 45.5%. These climatic conditions were harsher than those in which *A.* sec. *Terrei* was predominantly found. Under these conditions, *A.* sec. *Nigri* emerged as the most frequently recovered species from Alandan, Shurmast, and Abbasabad. The average temperature for air, soil, and water were recorded at 29.6 °C, 23.2 °C, and 23.7 °C, respectively, with a mean humidity of 49.3%. Nevertheless, since no significant differences were detected between the evaluated environmental parameters and fungal species abundance (*p* > 0.05), the distribution of fungal species across the lakes was not influenced by temperature and humidity. Tables 2 and 3 present detailed data on the influence of environmental factors on the distribution of *Aspergillus* species.

**Table 2:** Influence of environmental factors on *Aspergillus* species distribution during the first round of sampling (July 2023).

	**Environmental factors** *				**Fungal load (Average CFU /mL)**					
**Lake Name**	**Air temperature (°C)**	**Soil/Water temperature (°C)**	**Humidity (%)**	**Sample type**	***A.* sec. *Flavi***	***A*. sec. *Fumigati***	***A.* sec. *Nigri***	***A*. sec. *Terrei***	***A.* sec. *Nidulanti***	
Elimalat	33	23	31	Soil	2	2	22			First Round
				Sediment	2		2			
		25		Water			7			
Churet	27	24	52	Soil						
				Sediment			3			
		22		Water			2	3		
Alandan	38	28	25	Soil			15		5	
				Sediment			8			
		30		Water	2					
Shourmast	27	23	44	Soil	15		8			
				Sediment	8		40			
		25		Water			23			
Abbasabad	36	33	52	Soil	2		23	7		
				Sediment			8	2		
		32		Water			10			
Avidar	33	27	38	Soil			32			
				Sediment			23			
		29		Water	2		18			
Jade Nezami	38	29	39	Soil	2	2	30			
				Sediment			18			
		31		Water		3	3			

* The distribution of fungal species across the lakes was not influenced by temperature and humidity (*P*>0.05).

**Table 3:** Influence of environmental factors on *Aspergillus* species distribution during the second round of sampling (September 2023).

	**Environmental factors** *				**Fungal load (Average CFU /mL)**					
**Lake Name**	**Air temperature (°C)**	**Soil/Water temperature (°C)**	**Humidity (%)**	**Sample type**	***A.* Sec. *Flavi***	***A.* sec. *Fumigati***	***A.* sec. *Nigri***	***A*. sec. *Terrei***	***A*. sec. *Nidulanti***	
Elimalat	20	13		Soil	2			212		Second Round
				Sediment	3	2	8	23		
		14		Water		2		2		
Churet	23	20	58	Soil	5		8	10		
				Sediment	5	2	9	52		
		18		Water	5		8			
Alandan	24	21	60	Soil	8	2	5	2		
				Sediment	13	2	27	12		
		20		Water	5		15			
Shourmast	21	17	50	Soil	2		27			
				Sediment			47	13		
		19		Water	7		30	2		
Abbasabad	31.6	17	65	Soil	1		5	1		
				Sediment	2		7			
		16		Water	3		7			
Avidar	21	14	56	Soil						
				Sediment	2	22	7	63		
		16		Water				7		
Jade Nezami	29	24	52	Soil	75		8	5		
				Sediment	22	2	9	3		
		24		Water	3		5			

* The distribution of fungal species across the lakes was not influenced by temperature and humidity (*P*>0.05).

### 3.3 Susceptibility profile

AFST was performed solely on the isolates that were obtained at least once from SDA media enriched with ITZ and VRZ. The cultured isolates may demonstrate high MICS against the antifungal agents; nevertheless, the AFST assay was carried out to confirm these results. A total of 190 isolates were recovered from either ITZ-SDA or VRZ-SDA. According to the M59 guideline, 5.2% (6/116 isolates) among *A.* sec. *Flavi*, 23.8% (5/21) among *A.* sec. *Fumigati,* and 4.1% (12/295) among *A.* sec. *Terrei* were non-wild types against ITZ. All isolates belong to *A.* sec. *Nigri* were reported as wild type strains. Totally, 23 (5.3%) of isolates were identified as ITZ-resistant *Aspergillus* species. In case of VRZ, 0.7% (2/273) of *A.* sec. *Nigri* and 23.8% (5/21) of *A.* sec. *Fumigati* were non-wild types against VRZ. Conversely, all strains of both *A.* sec. *Terrei* and also *A.* sec. *Flavi* were reported as wild-type against VRZ. Altogether, 7 isolates (approximately 1%) were identified as VRZ-resistant *Aspergillus* species. Hence, VRZ was more effective antifungal rather than ITZ. Table 4 shows the MIC values for all screened *Aspergillus* species grown on antifungal- supplemented SDA media.

**Table 4:** Antifungal susceptibility profile of *Aspergillus* species isolated from soil, sediment, and water samples during July and September 2023.

***Aspergillus* section species**	Antifungal Agent**	*MICs (μg/ml)														
		**0.032**	**0.063**	**0.125**	**0.25**	**0.5**	**1**	**2**	**4**	**8**	**16**	**Range**	**G-Mean**	**MIC**_**50**_	**MIC**_**90**_	**Mode**
**All section** **(N=190)**	**ITZ**		**8**	**13**	**58**	**70**	**20**	**4**	**1**		**16**	**0.063-16**	**0.406**	**0.5**	**1**	**0.5**
	**VRZ**		**5**		**39**	**102**	**37**	**1**	**5**	**1**		**0.063-8**	**0.507**	**0.5**	**1**	**0.5**
*A.* sec. *Nigri* (N=112)	ITZ		2	5	37	51	14	3				0.063-2	0.408	0.5	1	0.5
	VRZ		2		18	55	35	1	1			0.063-4	0.552	0.5	1	0.5
*A.* sec. *Flavi* (N=41)	ITZ		4	4	12	15	5	1				0.063-2	0.328	0.5	1	0.5
	VRZ		3		10	27	1					0.063-1	0.369	0.5	0.5	0.5
*A.* sec. *Terrei* (N=30)	ITZ		2	3	8	4	1				12	0.063-1	0.322	0.5	0.5	0.5
	VRZ				10	19	1					0.25-1	0.406	0.5	0.5	0.5
*A.* sec. *Fumigati* (N=7)	ITZ			1	1				1		4	0.125-16	3.623	-	-	16
	VRZ				1	1			4	1		0.25-8	2.208	-	-	4

* MIC: minimum inhibitory concentration; **Antifungal agents: ITZ; Itraconazole, VRZ; Voriconazole. The numbers in highlighted area represent the number of non-wild type strains for each certain species according to CLSI M59 ECV values.

## 4 Discussion

Coastal ecosystems, encompassing sandy beaches, lake shores, and adjacent aquatic environments, are increasingly recognized as potential reservoirs of microbial pathogens, including fungi, which may pose risks to human health. These environments become contaminated through human activities, animal waste, and organic debris, facilitating the dissemination of opportunistic pathogens such as *Aspergillus* species (21, 22). Despite this, epidemiological data linking fungal infections to coastal exposure remain limited, particularly in regions like Iran. This study is the first to investigate the diversity and antifungal susceptibility of *Aspergillus* spp. in the water and soil of lake shores in northern Iran, offering novel insights into the ecological and clinical implications of these fungi in freshwater coastal environments. The present study provides a comprehensive analysis of the mycobiome associated with recreational lakes in Mazandaran province, Iran, with a focus on the molecular identification, prevalence, and antifungal susceptibility profiles of *Aspergillus* species isolated from various substrates (dry soil, sediment, and water). Our results identified *A.* sec. *Terrei* as the most prevalent (41.37%) species. *A. terreus* belongs to the *Terrei* section is particularly significant due to its inherent resistance to amphotericin B and its unique immune interaction traits, which lead to poorer outcomes compared to *A. fumigatus* (23–25). In Iran, *A.* sec. *Flavi* has been identified as the main etiological fungal agent responsible for colonization or allergic/infectious respiratory diseases (26, 27). Furthermore, *A*. sec. *Flavi* was the most prevalent *Aspergillus* section among fungi isolated from the Caspian Sea coastline (22). In contrast, it is important to note that *A. terreus* is the most prevalent species in southern Iran, particularly along the Persian Gulf coastlines (12). The predominance of *A.* sec. *Terrei* in soil samples (273 CFU/mg) and *A.* sec. *Nigri* in water and sediment samples (105 CFU/mL and 176 CFU/mL) suggests substrate-specific adaptations, potentially driven by differences in moisture, organic matter, and microbial interactions. Furthermore, *A.* sec. *Nigri* was identified as the species most commonly found in lakes exhibiting the highest humidity index, in contrast to other locations. These findings are consistent with a previously environmental study reporting the ubiquitous presence of *Aspergillus* species in soil and aquatic ecosystems, where they thrive due to their metabolic versatility and resilience to environmental stressors (28). Elimalat, located in the western part of Mazandaran with richer vegetation, diverse water sources, and a more moderate climate, provides favorable conditions for fungal growth, which demand further investigation. Notably, the high prevalence of *A.* section *Terrei* in Elimalat Lake, a popular recreational site, underscores the influence of human activity on fungal distribution, as tourist-heavy areas may experience increased organic inputs from litter and waste. Both Elimalat and Churet lakes, located >1,000 m above the Caspian Sea, showed the highest rate of *A*. sec. *Terrei*; while in the case of Avidar, which is located 125 m above the Caspian Sea, *A.* sec. *Terrei* and *A.* sec. *Nigri* comprised equal rates. *A.* sec. *Nigri* was most frequently isolated from Abbasabad, Alandan, and Shurmast with almost equal rate across these lakes. *Aspergillus* sec. *Flavi* was the most frequently isolated section from Jadeh Nezami lake, which is very close to the urban area and located only 51 m above the Caspian Sea.

The WHO has classified *Aspergillus* species as high-priority pathogens due to their potential to cause opportunistic infections, particularly in immunocompromised individuals (29). Although direct epidemiological data linking lake exposure to fungal infections are scarce, the presence of potentially pathogenic *Aspergillus* species in areas frequented by visitors raises concerns. Immunosuppressed individuals, as well as those with chronic respiratory conditions or diabetes, are particularly vulnerable to aspergillosis following inhalation of fungal conidia (30). The detection of *A*. sec. *Fumigati*, a known etiological agent of invasive aspergillosis, in water and sediment samples (35 CFU/mL) is particularly alarming, as water-based activities such as swimming may facilitate exposure to aerosolized conidia (31). Furthermore, the variation in fungal prevalence across lakes, with Elimalat showing the highest may reflect differences in water quality, soil composition, temperature, and anthropogenic pressures. Elimalat, located in the western part of Mazandaran with richer vegetation, diverse water sources, and a more moderate climate, provides favorable conditions for fungal growth, which demand further investigation.

A critical aspect of this study, with significant implications for clinical management and environmental health, was the identification of *Aspergillus* isolates exhibiting high MICs against antifungal agents. In total, 23 (5.3%) of the isolates were recognized as *Aspergillus* species with high MICs against ITZ, with *A.* sec. *Terrei* showing the highest rate of non-wild type species (12/295, 4.1%). This pattern is concerning, as ITZ is the main drug of choice for the empirical therapeutic management for aspergillosis in Iran (32). The high resistance rate in *A.* sec. *Terrei* is particularly troubling, given its clinical relevance and association with severe infections and also intrinsic resistance to amphotericin B. Global reports have linked the emergence of azole-resistant *Aspergillus* strains to environmental factors, such as the widespread use of azole fungicides in agriculture (33, 34). Although our study did not explore the specific drivers of resistance, the presence of isolates with high MICs in recreational lakes suggests a potential environmental reservoir for resistant strains, highlighting the need for further research into local agricultural practices and their impact on fungal populations.

Human behaviors, such as littering and inadequate waste disposal, can exacerbate fungal contamination by altering the native mycobiome and providing substrates for fungal growth. Similar to the coastal environmental studies, our results indicate that soil substrates harbor higher fungal loads than water, likely due to the stability of sediments as a niche for fungal proliferation. The summer sampling period, coinciding with peak tourist activity in Mazandaran, may have increased fungal contamination as a result of increased human presence and organic waste. To reduce these risks, public health strategies should focus on improving hygiene at recreational lakes. The WHO recommends including microbial contamination in environmental surveys and promoting hygiene awareness among visitors (35, Volume 1). Practical steps, such as providing waste disposal, designating non-swimming areas, and educating the public on personal and environmental hygiene, could significantly lower fungal exposure. Regular monitoring of recreational waters could also guide interventions to protect public health (5, 35, Volume 1).

This study is subject to several important limitations. First, due to resource constraints, species-level identification of *Aspergillus* isolates was not possible, as advanced molecular techniques such as sequencing were unavailable. Such methods could enhance the resolution of fungal diversity and pathogenicity, providing a clearer picture of the health risks caused by specific species. Second, sampling was restricted to summer 2023, limiting assessment of seasonal variations in fungal prevalence and resistance patterns. Multi-seasonal sampling reveal temporal dynamics in the mycobiome, particularly in response to climatic and anthropogenic factors (30). Finally, this study did not investigate the environmental or agricultural factors contributing to antifungal resistance, which could provide insights into the broader ecological context of our findings. Future research should address these gaps by employing high-throughput sequencing, conducting year-round sampling, and exploring the interaction between environmental fungicide use and resistance development.

## 5 Conclusion

This study highlights the widespread presence of *Aspergillus* species in the recreational lakes of Mazandaran, with significant antifungal resistance patterns that pose potential risks to public health. The diversity and prevalence of *Aspergillus* sections, coupled with resistance to key antifungals, underscore the need for vigilant monitoring of environmental fungal populations. Sampling restricted to a single season (summer 2023) prevented evaluation of seasonal variability. Section-level identification also restricted ecological and clinical specificity. By integrating fungal contamination into public health frameworks and promoting sustainable management practices, policymakers and communities can mitigate the risks associated with recreational lake use. Raising awareness among visitors, establishing standardized sanitation protocols, and conducting further research into the ecological drivers of fungal resistance are critical steps toward ensuring the safety and sustainability of these vital recreational areas.

## Data Availability

The raw data supporting the conclusions of this article will be made available by the authors, without undue reservation.
